# Genotoxin-producing *Salmonella enterica* induces tissue-specific types of DNA damage and DNA damage response outcomes

**DOI:** 10.3389/fimmu.2023.1270449

**Published:** 2024-01-11

**Authors:** Maria Lopez Chiloeches, Anna Bergonzini, Océane C. B. Martin, Nicole Bergstein, Saskia F. Erttmann, Kyaw Min Aung, Nelson O. Gekara, Javier F. Avila Cariño, Ioannis S. Pateras, Teresa Frisan

**Affiliations:** ^1^ Department of Molecular Biology and Umeå Centre for Microbial Research (UCMR) Umeå University, Umeå, Sweden; ^2^ Biological and Medical Sciences Department, University Bordeaux, Centre National de la Recherche Scientifique (CNRS), Institut de Biochimie et Génétique Cellulaires (IBGC), Unité Mixte de Recherche (UMR) 5095, Bordeaux, France; ^3^ Infection Oncology Unit, Institute of Clinical Molecular Biology, Christian-Albrechts University of Kiel, Kiel, Germany; ^4^ Department of Molecular Biosciences, The Wenner-Gren Institute, Stockholm University, Stockholm, Sweden; ^5^ Institute of Medical Microbiology and Hygiene, Medical Center - University of Freiburg, Faculty of Medicine, Freiburg, Germany; ^6^ Second Department of Pathology, “Attikon” University Hospital, Medical School, National and Kapodistrian University of Athens, Athens, Greece

**Keywords:** bacterial genotoxin, DNA damage response, inflammation, tissue specificity, inflammasome

## Abstract

**Introduction:**

Typhoid toxin-expressing *Salmonella enterica* causes DNA damage in the intestinal mucosa *in vivo*, activating the DNA damage response (DDR) in the absence of inflammation. To understand whether the tissue microenvironment constrains the infection outcome, we compared the immune response and DDR patterns in the colon and liver of mice infected with a genotoxigenic strain or its isogenic control strain.

**Methods:**

*In situ* spatial transcriptomic and immunofluorescence have been used to assess DNA damage makers, activation of the DDR, innate immunity markers in a multiparametric analysis.

**Result:**

The presence of the typhoid toxin protected from colonic bacteria-induced inflammation, despite nuclear localization of p53, enhanced co-expression of type-I interferons (*IfnbI*) and the inflammasome sensor *Aim2*, both classic features of DNA-break-induced DDR activation. These effects were not observed in the livers of either infected group. Instead, in this tissue, the inflammatory response and DDR were associated with high oxidative stress-induced DNA damage.

**Conclusions:**

Our work highlights the relevance of the tissue microenvironment in enabling the typhoid toxin to suppress the host inflammatory response *in vivo.*

## Introduction

The typhoid toxin (TT) is produced by *Salmonella enterica* serovars Typhi, Paratyphi, and *S. enterica* subspecies *arizonae*, *diarizonae*, and *javiana* (1–4). TT is a tripartite bacterial genotoxin with two enzymatically active moieties (PltA and CdtB) and a binding PltB subunit that forms a homopentameric ring. PltA has been identified as an ADP-ribosyl transferase with as yet unknown cellular target(s), while CdtB acts as a DNase, sharing conserved residues with the mammalian deoxyribonuclease (DNase) I (1, 5, 6). The CdtB catalytic subunit induces DNA cleavage and activates the classical DNA damage response (DDR) in intoxicated cells (1, 7, 8). Mutation of the PltA catalytic site (PltA^E133A^) does not affect the activity of the typhoid toxin, suggesting that CdtB, through induction of DNA damage, is the primary driver of cellular toxicity *in vitro* (9).

The CdtB subunit is also present in another class of bacterial genotoxins, namely the cytolethal distending toxin (CDT) family, produced by several Gram-negative bacteria [reviewed in (10)].

Infection with TT- or CDT-producing bacteria induces DNA breaks, which activate DDR. Intoxicated cells arrest in the G1 and/or G2 phase of the cell cycle to repair the damaged DNA (11–16). However, if the damage is too extensive, cells predominantly enter a permanent, quiescent state known as senescence (8, 17). Senescent cells secrete a plethora of pro-inflammatory mediators, such as IL1, IL6, IL8, and metalloproteases MMP-1 and MMP-3, which is known as senescence-associated secretory phenotype (SASP) (18, 19). DNA-damage-dependent inflammation can also be induced by leakage of DNA into the cytosol. DNA fragments can be sensed by specific receptors such as the cyclic GMP-AMP synthase (cGAS), which triggers activation of the interferon regulatory factor (IRF3) and the Nuclear Factor κB (NFκB), resulting in the production of pro-inflammatory cytokines, such as type-I interferons (IFN-Is), IL1 and IL6 (20). One of the transcriptional targets of IFN-Is is the inflammasome sensor for cytosolic DNA, called Absent in Melanoma 2 (AIM2), that mediates the catalytic cleavage of IL1 family member cytokines such as IL1β and IL18 via activation of caspase-1 and promotes pyroptosis and inflammation (21, 22).

In light of the inflammatory properties of DNA damage, it is still not understood why bacteria produce DNA-damaging toxins. Previous studies have shown that the inflammatory effects of infection with TT-producing *Salmonella* are tissue-dependent (23, 24). Infection exerted an anti-inflammatory effect in the small and large intestine, characterized by lack of leukocyte infiltration, presence of alternatively activated M2-like macrophages, higher levels of T regulatory cells, and an enhanced ratio of anti- to pro-inflammatory cytokines compared with mice infected with a strain lacking genotoxigenic activity. Conversely, similar levels of neutrophil infiltration were observed in the liver, independent of the bacterial strain (23).

To understand whether the tissue microenvironment affects the host immune response to the genotoxigenic *Salmonella*, we compare the innate immune and DDR response in liver and colon of mice infected with a *S.* Typhimurium strain expressing the functional typhoid toxin (herein named MC1 TT) with an isogenic *Salmonella* control strain lacking the CdtB subunit (herein named MC1 Δ*cdtB*) for 10 days (23). Our data emphasize the importance of the microenvironment to determine the outcome of infection with genotoxigenic *Salmonella* and tackle underexplored mechanisms of how genotoxin-producing bacteria may hijack mechanisms devoted to the maintenance of the colonic homeostasis in response to DNA damage to promote a successful infection.

## Materials and methods

### Infection

129S6/SvEcTac female mice were housed in a pathogen-free facility. At 8 weeks old, mice were orally infected with *Salmonella* strains at a dose of 10^8^. At 10 days post-infection, colons and livers were collected from mice as previously described (23, 24). Briefly, the distal part of the colon was prepared as Swiss rolls, and the central part of the liver with the gallbladder was collected for histologic analysis. Tissues were embedded in cryomolds containing Tissu-Tek OCT (Sakura Finetek, Tokyo, Japan), immediately placed on dry ice, and stored at -80°C until sectioning. The remaining parts of the organs were homogenised in phosphate buffer saline (PBS) and plated on LB plates supplemented with chloramphenicol (50 μg/ml) to assess bacterial recovery. The Regional Animal Studies Ethical Committee Northern Norrland, Sweden, reference numbers A17-17 and A23-21, approved this study protocol.

### Irradiation

At 12 weeks old, mice were exposed to a single sublethal dose of 9 Gy (total body irradiation) using a Gammacell 40 irradiator (MDS Nordion, Ottawa, ON, Canada, now provided by Best Theratronics Ottawa, ON, Canada) with Cesium-137 as the source. Radiation was given as a dose of 1Gy per min for 9 minutes. The mice were euthanised 12 hours post-irradiation, and colons and livers were collected as described above. The Regional Animal Studies Ethical Committee Northern Norrland, Sweden, reference number A25-19, approved this study protocol.

### Cell lines

The human colonic epithelial cell lines 1CT were previously described (25). 1CT cells were cultured in complete medium, composed as follows: high‐glucose Dulbecco’s Modified Eagle (DMEM) medium/medium 199 (4530, Sigma‐Aldrich Merck, Darmstadt, Germany, at ratio 4:1), supplemented with 2% fetal bovine serum (FBS; 10270-106, Gibco ThermoFisher Scientific, Waltham, MA, USA), epidermal growth factor (EGF; 20 ng/ml, E9644, Sigma‐Aldrich Merck), hydrocortisone (1 mg/ml, H0396, Sigma‐Aldrich Merck), insulin (10 mg/ml, I9278, Sigma‐Aldrich), transferrin (2 mg/ml, T0665, Sigma‐Aldrich Merck), sodium selenite (5 nM, S5261, Sigma‐Aldrich Merck), and geneticin (G418 sulfate) sulfate (50 μg/ml, G1397, Sigma‐Aldrich Merck).

Human colonic fibroblasts (CRL-1459, ATCC, Manassas, VA, USA) were maintained in Eagle’s Minimum Essential Medium (EMEM; 30-2003, ATCC, Manassas, VA, USA) supplemented with 10% of FBS (10270-106, Gibco, ThermoFisher Scientific) and 10 μg/ml of ciprofloxacin (17850, Sigma‐Aldrich Merck).

### Peripheral blood mononuclear cells isolation and monocyte purification

Human PBMCs were obtained from healthy donors (Blood Bank, Umeå University Hospital) using the standard procedure of Lymphoprep centrifugation (07801, STEMCELL Technologies, Vancouver, British Columbia, Canada). The recovered cells were counted by Trypan Blue (15250-061, Gibco ThermoFisher Scientific) exclusion, revealing a routine viability of more than 99%. CD14-positive cells were isolated from PBMCs using the EasySep™ Human CD14 Positive Selection Kit II (17858, STEMCELL Technologies) following the manufacturer’s instructions. The Swedish Ethical Review authority approved the use of the donors’ blood, Ethical permit 2021-00913.

### Immunocompetent organotypic 3D tissue model

Colonic tissue models were established as previously described (26). On day 5, 2x10^6^ PBMCs or 4x10^5^ monocytes were resuspended in 150μl of RPMI 1640 (R8758, Sigma‐Aldrich Merck) supplemented with 10% FBS (10270-106, Gibco, ThermoFisher Scientific) and added to the collagen matrix. Cells were incubated for two days at 37°C in 5% CO_2_ to allow sedimentation into the matrix. On day 7, 6x10^5^ 1CT cells were resuspended in 150μl DMEM complete medium (D6429, Sigma‐Aldrich Merck, Darmstadt, Germany) without antibiotics, overlaid on the fibroblast/collagen matrix and incubated for 4 hours at 37°C in 5% CO_2_, after which 2 ml complete DMEM medium without antibiotics was gently added to the insert. The models were cultivated for 24 hours at 37°C in 5% CO_2_ before infection.

Human organotypic colonic models were infected at a MOI of 25:1 as previously described (26). After infection, 2 ml of RPMI 1640 (R8758, Sigma‐Aldrich Merck) supplemented with 10% FBS (10270-106, Gibco, ThermoFisher Scientific) and gentamicin (100μg/ml, Sigma‐Aldrich Merck) was added into each well to kill extracellular bacteria and incubated for 1 hour. The “killing medium” was then changed to “maintaining medium,” RPMI supplemented with 10% FBS and 10μg/ml gentamicin, for three additional days to avoid overgrowth of bacteria. On day 3 post-infection, 2 ml of 2M sucrose solution was added to the inner and outer chambers, and samples were incubated for 1 hour at room temperature (RT). Subsequently, the models were embedded in Tissue-Tek OCT (Sakura Finetek, Tokyo, Japan), snap-frozen, and stored at -80°C. Ten micrometer sections were cut at -20°C using the cryostat Leica CM3050S, and slides were stored at -80°C until immunofluorescence analysis.

Hematoxylin and eosin staining was employed to assess the morphology of the organotypic 3D tissue models. Briefly, slides were fixed for 20 minutes in 4% paraformaldehyde (PFA; 1004965000, Sigma‐Aldrich Merck) at RT. After fixation, the samples were washed three times for five minutes in PBS under gentle shaking. The slides were then dipped twice into hematoxylin solution (GHS216, Sigma‐Aldrich Merck) for 3 seconds and rinsed in three different baths of tap water to remove unbound dye. The samples were then submerged in an eosin Y solution (HT110116, Sigma‐Aldrich Merck) and afterward dipped once into 95% and into 99% ethanol solutions to allow the dehydration of the tissues. Finally, the slides were mounted in 40% glycerol, covered with coverslips, and stored at +4°C until imaging.

### Flow cytometry

Flow cytometry analysis was performed by direct immunofluorescence analysis using the anti-human MultiMix™ Dual-Colour labeled with fluorescein isothiocyanate (FITC) (FR700, Dako, Glostrup, Denmark) and R-phycoerythrin (RPE) (FR866, Dako, Glostrup, Denmark) directed against CD45 (leukocyte marker), CD3 (T lymphocytes marker) or CD19 (B lymphocytes marker), and CD14 (monocytes marker). Unstained cells and cells labeled with the respective isotype controls (X0932, Dako, Glostrup, Denmark) were used as negative controls. A minimum of 1x10^4^ cells were analyzed using BD Accuri C6 Plus System (Becton and Dickinson, Franklin Lakes, NJ, USA) equipped with 488- and 640-nm lasers, and Accuri C6 Plus software was used to analyse the data.

### Immunofluorescence

#### Mouse tissue

Organs embedded in OCT medium were sectioned at 6µm thickness with a Cryostat CM-3050S (Leica Microsystems, Wetzlar, Germany), mounted on SUPERFROST® Plus Microscope Adhesion Slides (10149870, ThermoFisher Scientific) and stored at -80°C until use. Sequentially cut sections were used for the immunofluorescence analysis.

IF staining of colonic and liver sections was done as described in (27) with modifications. Briefly, tissue fixation was done in either 4% PFA (1004965000, Sigma‐Aldrich Merck, for CD45 and γH2AX) or cold acetone (17124, Sigma‐Aldrich Merck, for F4/80-CD206 and p53) for 15 minutes. After fixation, tissues were washed twice in PBS under gentle agitation and permeabilised for 30 minutes in a humidified chamber at RT. Specific solutions for permeabilization were used for different immunostainings. Specifically, 1% BSA, 0.1% Triton X-100, and 2% FBS in PBS for CD45 and F4/80-CD206 co-staining; 3% BSA and 0.2%Triton X-100 in PBS for γH2AX; 1% BSA 0.1% Triton X-100 and 2% FBS in 5% normal goat serum (NGS) for p53. Tissues were further incubated with blocking solution (1% BSA, 2% FBS in PBS, or 5% NGS) for 30 minutes at RT in a humidified chamber. Following the blocking step, tissues were incubated with the corresponding primary antibody for 1 hour RT for rabbit anti-CD45 (1:100, ab10558, Abcam) and rat anti-F4/80 (1:100, MCA497G, BioRad) or overnight at +4°C for goat anti-CD206 (1:500, AF2535, R&D Systems), rabbit anti-γH2AX (1:200, 9718S, Cell Signalling) and rabbit anti-p53 (1:500, P53-CM5P-L, Leica Microsystems). After two sequential washings in PBS under gentle shaking, tissues were incubated with the appropriate Alexa-conjugated secondary antibody for 1 hour at RT (1:1000 in blocking buffer), rewashed in PBS, and mounted with DAPI/VECTASHIELD® (H-1200-100, Vector Laboratories, Newark, CA, USA) for counterstaining of nuclei. Images were acquired using a fluorescent Nikon 90i Eclipse microscope equipped with a CCD camera (DS, F11 Hamamatsu, Nikon, Tokyo, Japan). At least 2000 cells per mouse were analysed in ImageJ.

Oxidized DNA was detected as previously described (28). Briefly, tissue sections were incubated with RNase A (100 μg/ml, EN0531, Thermo Scientific) for 1 hour at 37°C followed by treatment with Proteinase K (10 μg/ml, P6556, Sigma‐Aldrich Merck) for 10 minutes. Samples were incubated with 2M HCl for 5 minutes to unwind DNA and neutralised with 1M Tris-base for 7 min. To block the nonspecific binding of the primary antibody, samples were incubated for 1 hour in Goat F(ab) Anti-Mouse IgG H&L in 10% BSA (1:100, ab6668, Abcam). Samples were then incubated with mouse anti-8-oxoG antibody (1:100, 12501, QED Bioscience) overnight, followed by incubation with an anti-mouse Alexa 647 conjugated-IgG2b secondary antibody (A21242, Invitrogen). Images were acquired with a confocal scanning microscope (Leica TCS SP8, Leica Microsystems). The number of positive cells and cell fluorescence intensity of individual positive cells were analysed in 5 different micrographs for each mouse in Image J.

#### Organotypic 3D model

Tissue sections were fixed in 4% PFA in PBS for 20 minutes at RT. Tissues were washed three times in PBS and incubated in a blocking buffer (0.25% Triton X-100 and 3% BSA in PBS) for 30 minutes at RT to allow permeabilization and avoid non-specific binding. The slides were further incubated for an additional blocking step in 10% normal goat serum (50197Z, Life Technologies) for 10 minutes at RT.

The following primary antibodies were used: rabbit anti-phosphorylated KAP1 (1:100, A300-767A, Bethyl laboratories Inc, TX, USA), rabbit anti-γ-H2AX (1:200, 9718S, Cell Signalling Technology), and mouse antibodies anti-53BP1 (1:100, BD612523, BD Transduction Laboratories) and anti-CD3 (1:50, M7254, Dako, Glostrup, Denmark). All primary antibodies were diluted in blocking buffer and incubated overnight at +4°C in a humid chamber. The tissues were then incubated with the corresponding Alexa-conjugated secondary antibodies for 1 hour at RT (1:1000 in blocking buffer). Images were acquired with a fluorescence microscope (Nikon 90i Eclipse microscope) equipped with software NIS-E AR and with a CCD camera (DS, F11 Hamamatsu, Japan) and a confocal scanning microscope (Leica TCS SP8, Leica Microsystems). The images were then examined using ImageJ software, and at least 100 nuclei per slide were counted. Cells were considered positive for phosphorylated-KAP1 and γ-H2AX when the mean fluorescence intensity was higher than the mean fluorescence intensity of uninfected cells plus two standard deviations. Alternatively, cells were counted positive for 53BP1 when nuclei showed ≥5 foci/cell.

### RNAscope™

For the *in-situ* detection at mRNA levels of *Aim2, Il10, Ifnb1, Ifng, Nlrp3*, and *Salmonella enterica fljB*, we used an RNAscope® Multiplex Fluorescent Reagent Kit (323100, Bio-techne, Oxford, UK) and performed the assay according to the manufacturer’s instructions. A positive ready-to-use probe targeting the housekeeping genes *Polymerase II Subunit A (Polr2a), Peptidylpropyl isomerase B (PPIB)* and *Ubiquitin C (UBC)* (320881, Bio-techne) and a negative control probe targeting the *DapB* mRNA from the *Bacillus subtilis* strain SMY (320871, Bio-techne) were used to assess the mRNA quality of the samples.

Following fixation and dehydration, tissue sections were pre-treated with Protease IV (Bio-techne) for 30 minutes at RT and incubated with the corresponding probe mix for 2 hours at 40°C. After subsequent steps for signal amplification, tissue sections underwent two washing steps of 2 minutes with washing buffer provided by Bio-techne at RT under gentle agitation. The signal from target and control probes was detected after incubation with the fluorescent dyes Opal 520, Opal 570, and Opal 690 (1:750 for target probes and 1:1500 for control probes, NEL810001KT, PerkinElmer, Waltham, MA, USA). All incubation steps were done at 40°C in the HybEZ™ II Oven (321719, Bio-techne). Nuclei were counterstained and mounted with DAPI/VECTASHIELD® (H-1200-100, Vector Laboratories). Images were acquired with a confocal scanning microscope (Leica TCS SP8, Leica Microsystems). The expression levels of each target probe were quantitatively evaluated using the H-score, as recommended by the manufacturer.

### RNAscope™ combined with IF

For the co-detection of target mRNA probes (*Salmonella enterica fljB*) and protein (γH2AX), we used the RNA-Protein Co-Detection Ancillary Kit (323180, Bio-techne), following manufacturers’ instructions. Briefly, tissue sections were fixed in pre-chill 4% PFA (Sigma‐Aldrich Merck) for 15 minutes at +4°C and dehydrated in sequential immersions of 50%, 70%, and 100% ethanol. Tissue sections were further incubated with the corresponding primary antibody overnight at +4°C, followed by a washing step in 0.1% Triton X-100 in PBS and post-primary fixation in 4% PFA (Sigma‐Aldrich Merck) for 30 minutes at RT. Sections were then pre-treated with Protease IV for 30 minutes at RT and incubated with the corresponding probe mix for 2h at 40°C. Subsequent hybridization steps and probe signal detection with fluorescent dyes were done as described in the previous section. This was followed by incubation with the appropriate secondary antibody to detect the protein of interest for 30 minutes at RT in a humidified chamber. Sections were counterstained with DAPI/VECTASHIELD® (H-1200-100, Vector Laboratories). Images were acquired with a confocal scanning microscope (Leica TCS SP8, Leica Microsystems).

### Statistical analysis and principal component analysis

All the statistical analyses were performed using Prism 7 and Graph Pad Software. The significance of the difference between the three experimental groups was determined by ANOVA with Tukey’s post-test. *p*-values <0.05 were considered significant; * *p* < 0.05, ** *p* < 0.01, ***p < 0.001, **** *p* < 0.0001. If not indicated, differences were not statistically significant.

Principal component analysis (PCA) was performed to create an overview of all assessed parameters, investigate data integrity, identify potential outliers, and explore possible trends and groupings of the samples (29). All multivariate data analysis and model plots were performed in SIMCA 16.0 (Sartorius Stedim Data Analytics AB, Umeå, Sweden).

## Results

### Genotoxigenic *Salmonella* induces a different immune response in the liver and colon

**Figure 1:**
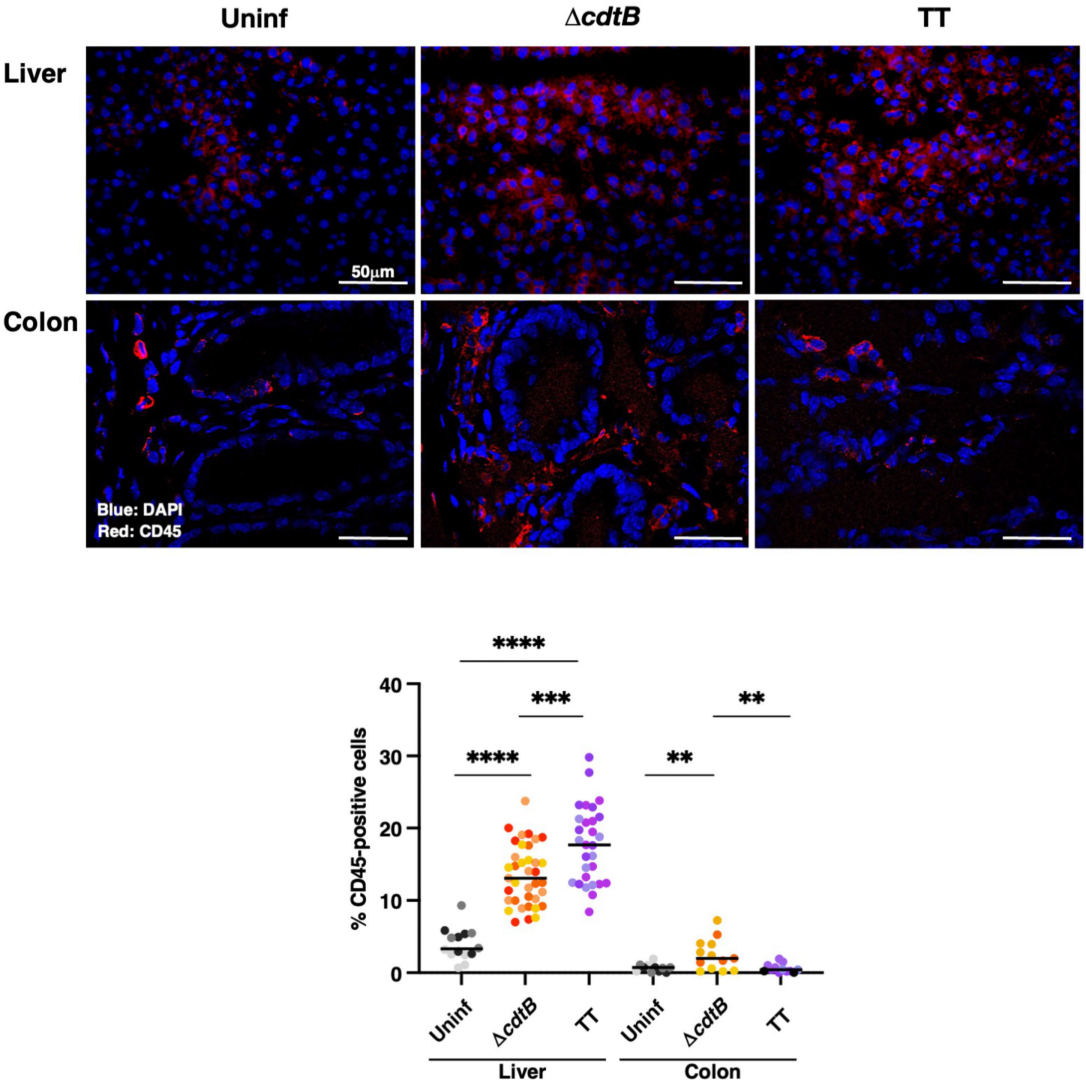
Infection with genotoxigenic *Salmonella* induces a different host immune response in the liver and colon. Mice were mock treated with PBS (Uninf) or infected with *Salmonella* MC1 Δ*cdtB* (Δ*cdtB*) or MC1 TT (TT) for 10 days, and the presence of leukocytes was detected by immunofluorescence with a rabbit anti-CD45 antibody in liver and colon. Upper panel: representative micrographs of the immunostaining targeting CD45-positive cells (red). Nuclei were counterstained with DAPI (blue). Lower panel: quantification of CD45-positive cells in both organs. The data are presented as percentage positive cells in all the micrographs acquired for each mouse. A different color shade identifies individual mice within a group. ** *p*-value ≤ 0.01; *** *p*-value ≤ 0.001; **** *p*-value ≤ 0.0001.

In the liver, increased levels of leukocytes (CD45 positive cells) were detected in all the infected animals compared to the uninfected group, with higher cell infiltration levels in mice infected with the genotoxigenic strain (**Figure 1**). Conversely, levels of leukocytes in the colon of mice infected with the MC1 TT strain were significantly lower than those observed in mice infected with the control strain MC1 Δ*cdtB* (**Figure 1**).

**Figure 2:**
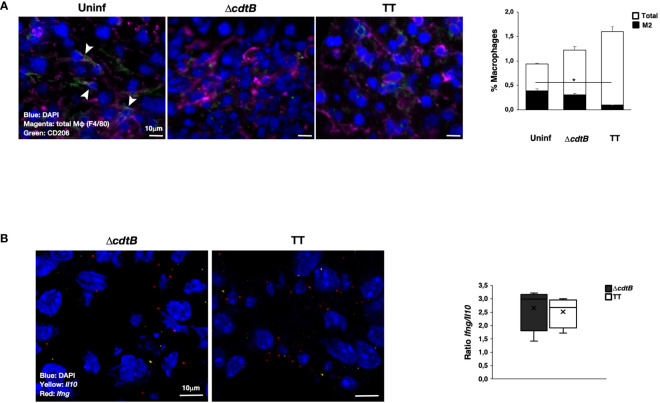
Infection with genotoxigenic *Salmonella* promotes an inflammatory response in liver. Mice were mock-treated with PBS (Uninf) or infected with *Salmonella* MC1 Δ*cdtB* (Δ*cdtB*) or MC1 TT (TT) for 10 days. **(A)** The presence of macrophages was detected using a rat anti-F4/80 antibody for total macrophages (magenta) and a goat anti-CD206 antibody for anti-inflammatory, M2-like macrophages (green). Nuclei were counterstained with DAPI (blue). Left panel: representative micrograph. Double-positive cells are indicated with white arrowheads. Right: quantification of F4/80-CD206-positive cells (mean ± SEM). * *p*-value ≤ 0.05. **(B)** mRNA levels expression of the pro-inflammatory cytokine *Ifng* (red) and the anti-inflammatory cytokine *Il10* (yellow) were assessed by RNAscope™. Nuclei were counterstained with DAPI (blue). Left panel: representative micrograph. Right panel: quantification of the level of mRNA expression. The data are presented as a ratio between the levels of *Ifng* mRNA and *Il10* mRNA. The sign “x” within the box plot represents the mean value.

In line with pronounced hepatic leukocyte infiltration, co-immunostaining with macrophage marker F4/80 and mannose receptor CD206, which identifies anti-inflammatory M2-like macrophages, showed a significant decrease in the number of M2-like macrophages recruited to the liver upon infection with genotoxigenic *Salmonella* (**Figure 2A**).

RNAscope™ was used to assess the mRNA levels of the pro-inflammatory cytokine *Ifng* and the anti-inflammatory cytokine *Il10* in the liver. Specific positive and negative probes were used to evaluate the quality and sensitivity of the assays (**Supplementary Figure S1**). Levels of *Il10* were lower than those observed for *Ifng* (**Supplementary Figure S2A**), and higher values of both mRNAs were present in mice infected with the genotoxigenic strain (**Supplementary Figure S2A**). However, no difference in the *Ifng*/*Il10* ratio was observed independent of the presence of the typhoid toxin (**Figure 2B**).

**Figure 3:**
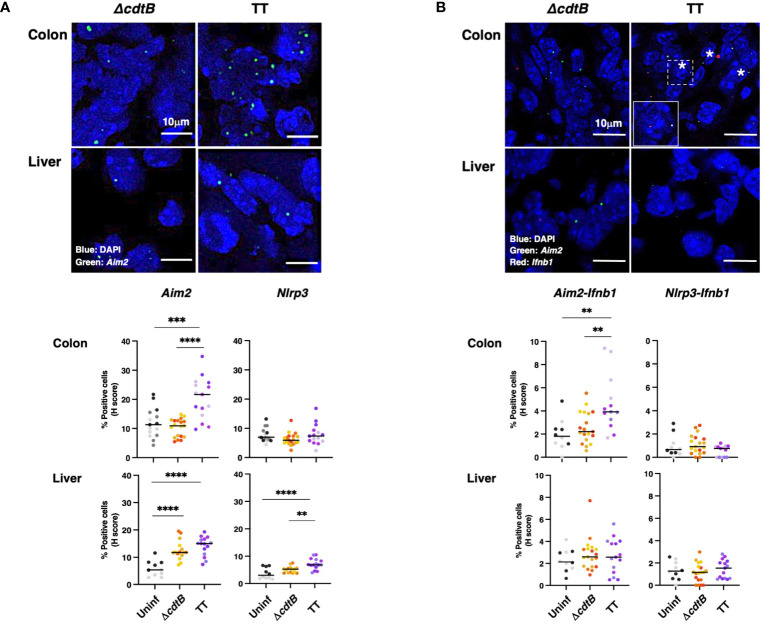
Tissue-specific expression of inflammasome sensor *Aim2* and co-expression with type-I interferon mRNA (*Ifnb1*) upon infection with genotoxigenic *Salmonella.* Mice were mock treated with PBS (Uninf) or infected with *Salmonella* MC1 Δ*cdtB* (Δ*cdtB*) or MC1 TT (TT) for 10 days. **(A)** The inflammasome sensors Aim2 and Nlrp3 expression levels were assessed in the colon and liver by RNAscope™. Upper panel: representative micrographs of *Aim2* (green) in *Salmonella*-infected groups in both organs. Nuclei were counterstained with DAPI (blue). Lower panel: quantification of *Aim2*- or *Nlrp3*-positive cells in the colon and liver. The data are presented as percentage positive cells in all the micrographs acquired for each mouse. A different color shade identifies individual mice within a group. ** *p*-value ≤ 0.01; *** *p*-value ≤ 0.001; **** *p*-value ≤ 0.0001. **(B)** Evaluation of co-expression of the inflammasome sensors *Aim2* or *Nlrp3* and *Ifnb1* in the colon and liver. Upper panel: representative micrographs of *Aim2* (green) and *Ifnb1* (red) in *Salmonella*-infected groups in both organs. Nuclei were counterstained with DAPI (blue). Asterisks indicate double-positive cells. Inset: higher magnification of the region marked with a dotted square. Lower panel: quantification of cells co-expressing *Aim2* or *Nlrp3* and *Ifnb1*, respectively, in the different experimental groups in the colon and liver. The data are presented as percentage positive cells in all the micrographs acquired for each mouse. A different color shade identifies individual mice within a group. ** *p*-value ≤ 0.01.

To evaluate tissue differences in the host response to the genotoxigenic strain, we assessed the mRNA levels of inflammasome sensors, which are key effectors in driving inflammation (30). We focused on Absent in Melanoma 2 (*Aim2*) and the nucleotide-binding oligomerization domain-like receptor (NLR) *Nlrp3*. These sensors were selected due to their activation by different stimuli: AIM2 is activated by cytosolic double-strand DNA (22) and NLRP3 responds to intracellular damage (e.g., sensing of K^+^ efflux) (31). Upon infection, a toxin-dependent induction of *Aim2* mRNA was observed in the colon (**Figure 3A**) despite similar levels of bacterial recovery in the two infected groups (24). In the liver of infected mice, the enhanced *Aim2* expression was toxin-independent (**Figure 3A**). The infection did not significantly change *Nlrp3* expression in colonic tissue, while higher *Nlrp3* levels were detected in the hepatic tissue after infection with the MC1 TT strain (**Figure 3A**).

*Aim2* is an interferon-stimulated gene (ISG) (30). Therefore, we evaluated the co-expression of *Aim2* and *Ifnb1* mRNAs in the colon and liver. Significant co-expression levels of *Ifnb1* and *Aim2* mRNAs were observed exclusively in the colon of mice infected with the genotoxigenic strain. In contrast, *Ifnb1* and *Nlrp3* co-expression was not observed in any analyzed tissues or conditions (**Figure 3B**). A tendency for increased *Ifnb1* mRNA levels was present in the colonic tissue of infected animals (**Supplementary Figure S2B**). However, this trend was not observed in the liver samples (**Supplementary Figure S2B**).

### Genotoxigenic *Salmonella* induces different types of DNA damage in the colon and liver

**Figure 4:**
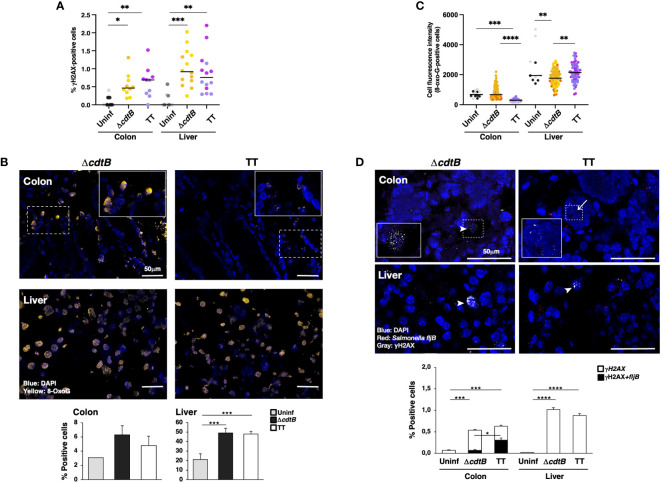
Tissue-specific proximity of DNA damage response marker and *Salmonella* upon infection with genotoxigenic *Salmonella*. Mice were mock treated with PBS (Uninf) or infected with *Salmonella* MC1 Δ*cdtB* (Δ*cdtB*) or MC1 TT (TT) for 10 days. **(A)** Quantification of γH2AX-positive cells by immunofluorescence in colon and liver. The data are presented as percentage positive cells in all the micrographs acquired for each mouse. A different color shade identifies individual mice within a group. * *p*-value ≤ 0.05, ** *p*-value ≤ 0.01; *** *p*-value ≤ 0.001. **(B)** Detection of 8-oxoG induced by oxidative damage by immunofluorescence analysis using a mouse anti-8-oxoG specific antibody. Upper panel: representative micrographs showing cells carrying oxidative-induced DNA damage (yellow). Nuclei were counterstained with DAPI (blue). Inset: higher magnification of the region marked with a dotted square. Lower panel: quantification of the percentage of 8-oxoG-positive cells. **(C)** Quantification of the cell fluorescence intensity for each 8-oxoG-positive cell in the colon and liver. The data are presented as percentage positive cells in all the micrographs acquired for each mouse. A different color shade identifies individual mice within a group. ** *p*-value ≤ 0.01, *** *p*-value ≤ 0.001; **** *p*-value ≤ 0.0001. **(D)** Quantification of γH2AX (detected with a rabbit anti-γH2AX antibody) and *Salmonella fljB* mRNA probe was performed by a combined protocol of immunofluorescence and RNAscope™ in the colon and liver of *Salmonella*-infected mice. Upper panel: representative micrographs showing γH2AX positive cells (grey) and the Salmonella *fljB* (red) presence. Nuclei were counterstained with DAPI (blue). Arrowheads indicate γH2AX-positive cells, and arrows indicate the proximity of the two markers. Inset: higher magnification of the region marked with a dotted square. Lower panel: quantification of γH2AX- and double-positive cells in colon and liver of *Salmonella*-infected mice. * *p*-value ≤ 0.05, *** *p*-value ≤ 0.001; **** *p*-value ≤ 0.0001.

Infection promoted a significant increase in cells positive for the DDR marker γH2AX (a phosphorylated form of histone H2AX at serine 139) in both tissues in a toxin-independent manner (**Figure 4A**). Since both oxidative-induced and TT-induced DNA damage promotes H2AX phosphorylation (7, 32, 33), we performed additional experiments to assess the type of DNA damage induced in the colonic and hepatic tissues upon infection.

Oxidative-induced DNA damage was measured by the detection of 8-dihydro-8-oxoguanine (8-oxoG) using immunofluorescence analysis. Low numbers of positive cells were present in uninfected tissues in both organs (**Figure 4B**). The fluorescence intensity of each positive cell was quantified, and enhanced fluorescence intensity was observed in the colon of mice infected with the control strain compared to mice infected with the genotoxigenic strain (**Figure 4C**), despite a similar percentage of 8-oxoG positive cells (**Figure 4B**). Conversely, in the liver, the cell fluorescence intensity was higher in the MC1 TT-infected mice compared to mice infected with the MC1 Δ*cdtB Salmonella* (**Figure 4C**). Interestingly, we observed that the few 8-oxoG-positive cells in the liver of uninfected mice had a higher fluorescence intensity than the levels observed in the colon (**Figure 4C**).

As a next step, we investigated whether there was a correlation between the presence of genotoxigenic *Salmonella* and DNA damage response by assessing the proximity of the bacterium to cells activating the DDR. We established a combined protein/mRNA protocol to detect the levels of γH2AX by immunofluorescence in combination with the mRNA probe specific for the flagellin subunit *fljB* of *S. enterica*. Significantly higher levels of *fljB* mRNA were detected in infected samples compared to uninfected tissues, independent of the *Salmonella* strain used, supporting the specificity of the probe for *Salmonella* and lack of cross-reactivity with the colonic microbiota (**Supplementary Figure S2C**). We found a higher percentage of γH2AX-positive cells in proximity to the flagellin subunit *fljB* upon infection with the MC1 TT bacterium in the colon. In contrast, this proximity was not observed in the colon of mice infected with the MC1 Δ*cdtB* strain or in infected liver samples (**Figure 4D**).

**Figure 5:**
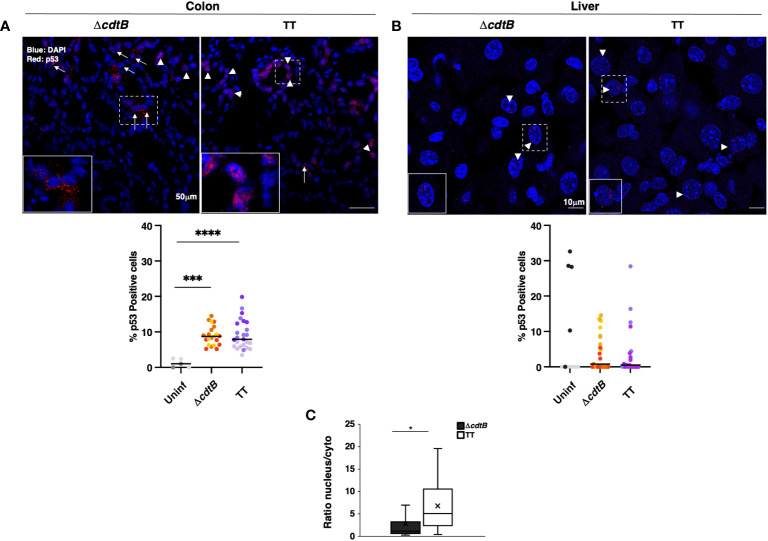
p53 tissue-dependent subcellular location upon *Salmonella* infection. Mice were mock-treated with PBS (Uninf) or infected with *Salmonella* MC1 Δ*cdtB* (Δ*cdtB*) or MC1 TT (TT) for 10 days. Levels of the p53 protein were assessed by immunofluorescence in the colon **(A)** and liver **(B)**. Upper panel: representative micrographs of organs showing differential localisation of p53. Arrowheads indicate nuclear p53 localization, and arrows indicate cytoplasmic distribution. Lower panel: quantification of p53-positive cells in colon **(A)** and liver **(B)** in the different experimental groups. The data are presented as percentage positive cells in all the micrographs acquired for each mouse. A different color shade identifies individual mice within a group. *** *p*-value ≤ 0.001, **** *p*-value ≤ 0.0001. **(C)** The data are presented as a ratio between the number of cells with nuclear versus the number of cells with cytoplasmic p53 distribution from panel **(A)**. The sign “x” within the box plot represents the mean value. **(A)** The “x” in the box plots represents the mean value. * *p*-value ≤ 0.05.

To complete the analysis of the DDR response, the levels of expression of the tumour suppressor protein p53 were investigated by immunofluorescence analysis. Higher percentage of p53-positive cells was detected in the colon of infected mice compared to the uninfected controls or hepatic samples (**Figures 5A, B**). However, p53 exhibited a nuclear localisation in the colonic tissue of mice infected with the genotoxigenic strain, while mice infected with the control strain showed a more dominant cytosolic p53 localization (**Figure 5C**). When detected in the liver, p53 exhibited an exclusively nuclear localization (**Figure 5B**).

To evaluate whether the intestinal immune response induced by genotoxigenic *Salmonella* was comparable to that caused by other agents that induce DNA breaks, we assessed the response produced by ionizing radiation (IR). We analyzed the infiltration of leukocytes and macrophages and the induction of γH2AX in the colon and liver of mice exposed to a single sublethal radiation dose of 9 Gy 12 hours post-treatment. We did not observe any signs of inflammatory response regarding leukocyte infiltration or changes in the proportion of M2-like CD206-positive macrophages in the colon or liver. However, differences in macrophage populations were observed, with a significant decrease in F4/80 positive cells in hepatic tissues upon irradiation (**Supplementary Figure S3**). As expected, significantly higher levels of γH2AX were present in both tissues upon irradiation. These data show that the presence of DNA breaks, neither in the colon nor in the liver, does not promote a strong inflammatory response within the analysed time.

### Principal component analysis

**Figure 6:**
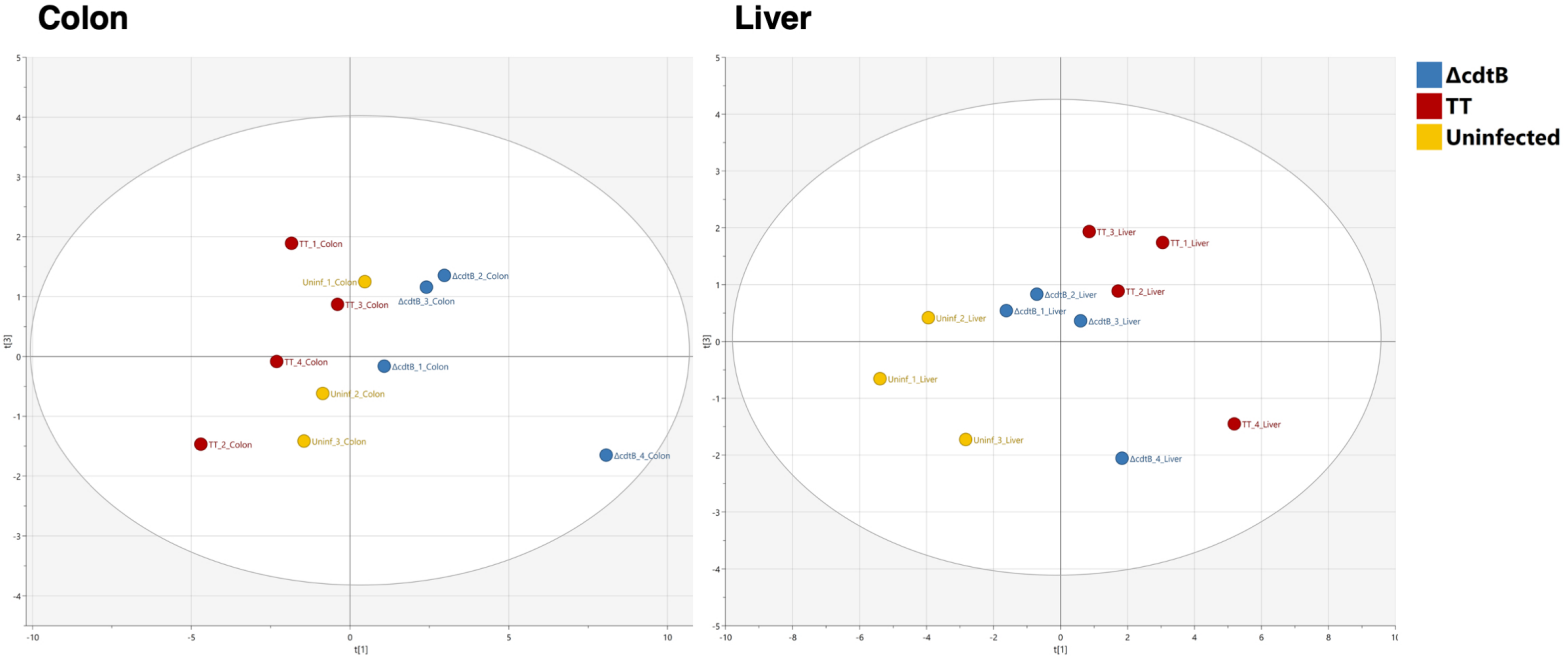
Principal Component Analysis. Unbiased Principal Component Analysis where the parameters presented in **Figures 1** to **4** from the colon (left) and the liver (right) have been analysed in an unsupervised manner.

An unbiased Principal Component Analysis was performed by combining all the parameters analysed in the study, including immune markers (CD45 for leukocytes, F4/80 for macrophages, CD206 for M2-like macrophages, *Ifng* and *Il10* mRNAs), markers for DNA damage (γH2AX, 8-oxo-G), and markers for DNA damage innate immune response (mRNA for type I interferon and the DNA-damage sensor AIM2). The PCA analysis shows that mice infected with the MC1 TT strain are well-separated from those infected with the isogenic MC1 Δ*cdtB* strain and clustered close to the non-infected mice in the colon (**Figure 6**). However, the pattern observed in the liver samples shows that both infected groups are clustered very closely and are well-separated from the uninfected group (**Figure 6**).

### Typhoid toxin-induced DDR is altered in an immunocompetent human organotypic 3D model

To assess whether the presence of immune cells influences the activation of DDR upon infection with the genotoxigenic strain in a human setting, we modified the organotypic 3D models previously developed in our laboratory (26) by embedding freshly isolated peripheral blood mononuclear cells (PBMCs) (2x10^6^ cells/model) or freshly purified monocytes (4x10^5^ cells/model) in the collagen matrix. Phenotypic characterization of the PBMC population, performed by flow cytometry analysis, showed that more than 90% of the cells were positive for the leukocyte marker CD45, with T lymphocytes (CD3 positive) representing the predominant population (62%), followed by monocytes (CD14 positive, 19%) and B lymphocytes (CD19 positive, 3.5%) (**Supplementary Figure S4A**). Upon isolation, 80% of the monocyte population was CD14 positive, with a small percentage (7.3%) of T lymphocytes (CD3 positive) (**Supplementary Figure S4A**).

**Figure 7:**
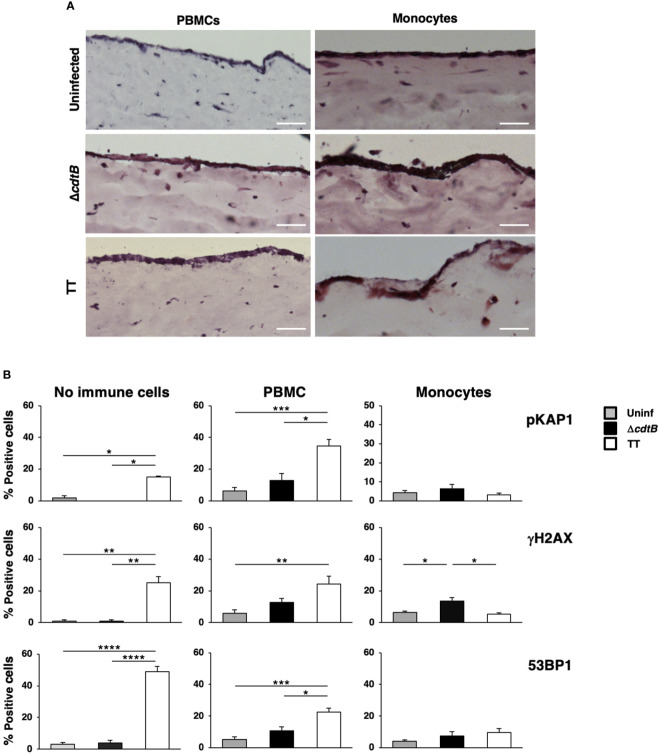
The presence of immune cells alters the toxin-induced DDR in a human organotypic 3D model. 1CT 3D culture models were established in the absence or presence of PBMCs or purified monocytes and left uninfected or infected with the MC1 TT or MC1 Δ*cdtB* strain at MOI 25:1 for 3 days. **(A)** Representative phase contrast micrograph of the 3D organotypic models stained with hematoxylin and eosin. **(B)** Induction of the DDR was assessed by immunofluorescence analysis, using antibodies specific for phosphorylated KAP1 (p-KAP1), phosphorylated H2AX (γH2AX), and 53BP1. Quantification of the cells positive for the DDR markers. Mean ± SEM of three independent experiments. * *p*-value ≤ 0.05; ** *p*-value ≤ 0.01; *** *p*-value ≤ 0.001; **** *p*-value ≤0.0001.

The numbers of cells embedded into the matrix were selected based on previous studies (34). The models were left uninfected or infected with the MC1 Δ*cdtB* or the MC1 TT for 72h to mimic an acute infection. **Figure 7A** shows that the addition of PBMCs did not significantly alter the model’s general architecture as assessed by Haematoxylin and Eosin (H&E) staining in uninfected or infected samples. However, in the presence of freshly isolated monocytes, the epithelial layer became more fragile upon infection and occasionally presented a multi-layered structure.

Activation of the DNA damage response was assessed by immunofluorescence analysis using markers for the phosphorylation of two early effectors of the DNA damage sensing machinery, KAP1 (pKAP1) and γH2AX (35, 36), and formation of 53BP1 foci at the site of the damaged DNA, which enables recruitment of repair effectors (37). As expected, we detected a significant increase of all the markers upon infection with the MC1 TT strain in the absence of immune cells (**Figure 7B**), confirming our previous results (26). In the presence of PBMCs, we still observed a toxin-induced increase of the DDR signature, but this was accompanied by increased levels of all the markers tested in uninfected and MC1 Δ*cdtB* infected cells (**Figure 7B** and **Supplementary Figure S4B**). This suggests that the addition of PBMCs promoted non-toxin-induced DNA damage, possibly due to induction of immune cell-induced oxidative damage. In contrast, the loss of a specific TT-induced DNA break-dependent response was associated with the presence of monocytes (**Figure 7B** and **Supplementary Figure S4B**).

These findings indicate that toxin-mediated DNA damage activates a default response to DNA-damaging agents in the colonic mucosa’s tolerogenic environment, suppressing *S.* Thyphimurium-induced inflammation. However, this effect is lost in the liver, where bacterium-induced inflammation is the predominant response.

## Discussion

We have demonstrated that the immune response to infection with the genotoxigenic *Salmonella* strain MC1 TT in the colon significantly differs from that induced in the liver. In the colon, despite detecting DNA strand breaks and subsequent activation of a DNA damage response, we observed a reproducibly sustained anti-inflammatory response (23, 24). Conversely, a potent inflammation was observed in the liver independent of the *Salmonella* strain.

The critical differences in colonic responses to infection with MC1 TT strain compared to the control strain were: i) elevated gene expression of the inflammasome sensor *Aim2* (**Figure 3**); ii) co-expression of *Aim2* and *Ifnb1* mRNAs (**Figure 3**); iii) the proximity of γH2AX-positive nuclei to the *Salmonella fljB* gene (**Figure 4**); iv) low levels of oxidative stress-induced DNA damage (**Figure 4**); and vi) a higher percentage of cells with nuclear p53 (**Figure 5**).

A different scenario was observed in the liver, where mice infected with both *Salmonella* strains presented: i) enhanced levels of inflammatory responses (**Figures 1** and **2**); ii) similar levels of *Aim2* upregulation in absence of co-localization with *Ifnb1* (**Figure 3**); iii) no proximity of the *fljB* mRNA to γH2AX-positive nuclei (**Figure 4**); iv) induction of oxidative-induced DNA damage; and v) a similar pattern of p53 induction (**Figure 5**).

### Microenvironment and modulation of host response

The anti-inflammatory effect exerted by the typhoid toxin may be dependent on the tolerogenic environment of the intestinal mucosa, characterised by the almost exclusive presence of M2-like macrophages under homeostatic conditions (uninfected mice) (24), compared to approximately 40% in the liver (**Figure 2**). The tolerogenic environment may cause an initial inhibitory effect, later supported by a sustained chronic production of type I IFNs, stimulated by the presence of cytoplasmic DNA fragments via the cGAS/STING/IFN-I axis (30, 38). Chronic production of type I IFNs promotes immunosuppression via diverse effects on immune cells (39), including inhibition of dendritic cells (DCs), with expansion and polarization of DCs subsets with an IL10-secreting suppressor phenotype; reduced IFNγ release from NK cells, an essential cytokine against intracellular bacteria such as *Salmonella* (40, 41); reduced recruitment of neutrophils, via inhibition of the chemokines CXCL1 and CXCL2; activation of myeloid-derived suppressor cells (MDSCs). This pattern is compatible with several observations we have made previously (24). We did not detect neutrophil influx, but a higher frequency of MDSC-induced cell types, such as M2-like macrophages and T regulatory cells (42). In addition, prolonged induction of type I IFNs due to toxin-dependent DNA damage may contribute to reduced levels of *Ifng* detected in the colon of mice infected with the genotoxigenic strain (23).

The role of immune microenvironment on the modulation of the toxin-induced DDR is supported by the organotypic model (**Figure 7**), where we observed a general increase of DDR markers in samples left uninfected or infected with the control strain MC1 Δ*cdtB* upon addition of the PBMCs. The observed effect may result from a PBMC-mediated inflammatory response and consequent production of DNA-damaging ROS. This observation aligns with a previous report showing that adding PBMCs promoted a 2- to 6-fold increase in pro-inflammatory cytokines IL6 and IL8 in a 3D oral mucosa model (34). Interestingly, the presence of myeloid cells promoted a loss of the toxin-induced DDR pattern associated with an alteration of the epithelial structure (**Figure 7**). These findings suggest that the host response to the bacterium is overwhelming in a non-tolerogenic environment, promoting tissue destruction, and masking the detection of the toxin-induced DDR. However, we cannot exclude that the different colonic and hepatic types of immune responses upon infection with the MC1 TT strain might be due to the 10-fold higher bacterial recovery in the liver (24), which may promote a more robust inflammatory response that covers the toxin-mediated DNA damage.

### DNA strand breaks and immune response

The absence of leukocyte influx in the colon and liver of mice exposed to a single dose of radiation (**Supplementary Figure S3**) indicates that the hepatic inflammatory response seen in mice infected with the genotoxigenic strain is mainly due to the bacteria-induced host response. The response observed in irradiated mice was unexpected but in agreement with previous results reporting a fast upregulation of the inflammatory cytokines and chemokines in rats with a dose of 25 Gy, associated with a transient increase of recruitment of neutrophils into the portal area but not into the hepatic parenchyma within a period of 3 to 6h post-irradiation (43). Thus, it is likely that, depending on the irradiation dose, the primary tissue response is to activate DNA repair, promoting a very limited, short-lived, and controlled inflammatory response to avoid further tissue damage, and that bacterial genotoxins hijack this response to ensure a stealth crossing of the intestinal barrier.

### Genotoxicity and AIM2 inflammasome

We observed that the upregulation of the inflammasome sensor *Aim2* was uncoupled from an increased inflammatory response in the colon of mice infected with the genotoxigenic strain (**Figure 3**). It has been previously shown that upregulation of AIM2 can activate a RalB-dependent autophagosome formation and recruitment of K63 polyubiquitinated AIM2 inflammasomes for degradation, resulting in reduced maturation of IL-1β β (44). This activity is independent of the inflammasome adaptor ASC and pro-inflammatory cysteine caspase-1 and is uncoupled from induced proinflammatory cell death. This negative feedback loop may explain why increased levels of *Aim2* do not correlate with an inflammatory response in the colon of mice infected with the genotoxigenic strain.

We observed enhanced *Aim2* levels in the liver of infected mice in the absence of co-expression with its inducer *Ifnb1* (**Figure 2**). Thus, AIM2 expression may be regulated in an IFN-I-independent manner. The nuclear factor E2-related factor-2 (Nrf2), a key transcription factor that regulates the cellular antioxidant responses, can promote AIM2 transcription (45, 46), which is consistent with the increased oxidative state associated with the bacteria-induced hepatic inflammation (**Figures 1** and **4**).

### Toxin-mediated DNA damage and p53

The product of the tumour suppressor gene *TP53* is a transcription factor activated in response to DNA damage. Stabilization of p53 determines the fate of damaged cells by inducing either cell cycle arrest, allowing DNA repair, or apoptosis promoting cell death (47). Inflammation is also associated with higher p53 levels, likely due to the production of ROS and subsequent oxidative-induced DNA damage (48, 49). Therefore, it is unsurprising that we observed significantly higher levels of p53 stabilization in the colon of mice infected with the control MC1 Δ*cdtB* strain (**Figure 5**), which aligns with the increased levels of γH2AX staining (**Figure 4**). This was also expected in the colonic tissue of mice infected with the genotoxigenic strain, which causes DNA fragmentation (24).

Interestingly, p53 showed prevalent nuclear localization in mice infected with the MC1 TT strain and a more dominant cytosolic distribution in mice infected with the control strain (**Figure 5**). The cytosolic fraction of p53 may regulate several processes, including autophagy and induction of ROS scavengers (50, 51). In the context of a strong inflammatory response observed in the colon of MC1 Δ*cdtB* infected mice, the cytosolic fraction of p53 may activate negative feedback mechanisms to control the bacterium and prevent irreparable tissue damage.

Surprisingly, we found very low levels of p53 stabilization in the liver of the infected mice in a strain-independent manner despite the strong inflammation and bacteria-induced DNA damage (**Figure 5**). One possible explanation is that the levels of p53 in inflamed colon and liver are orchestrated in a tissue-dependent manner by the presence of different cellular subsets and/or different patterns of chemokines/cytokines in a cell non-autonomous manner.

## Concluding remarks

This work highlights that the outcome of the DNA damage responses induced by infection with genotoxigenic *Salmonella* is tissue specific. This is somewhat surprising since DNA damage represents a substantial danger to the integrity of an organism. Yet, despite the activation of the DDR and increased abundance of cytosolic DNA sensors by the genotoxigenic *Salmonella*, the induction of an inflammatory response by fragmented DNA is prevented in the tolerogenic environment of the intestine, highlighting the role of the microenvironment in allowing the typhoid toxin to exert its activity as a suppressor of the host inflammatory response *in vivo*. The underlying molecular mechanisms of this effect remain elusive and will require immunologically competent *in vitro* models to be addressed. Our findings highlight a previously under-investigated gap in understanding how genotoxin-producing bacteria exploit host cellular defense responses to establish a successful infection.

## Data availability statement

The raw data supporting the conclusions of this article will be made available by the authors, without undue reservation.

## Ethics statement

The studies involving humans were approved by The Swedish Ethical Review authority. Ethical permit 2021-00913. The studies were conducted in accordance with the local legislation and institutional requirements. The human samples used in this study were acquired from a by- product of routine care or industry. Written informed consent for participation was not required from the participants or the participants’ legal guardians/next of kin in accordance with the national legislation and institutional requirements. The animal study was approved by Regional Animal Studies Ethical Committee Northern Norrland. The study was conducted in accordance with the local legislation and institutional requirements.

## Author contributions

ML: Conceptualization, Data curation, Formal analysis, Investigation, Methodology, Writing – original draft, Writing – review & editing. AB: Conceptualization, Formal analysis, Investigation, Methodology, Writing – review & editing. OM: Investigation, Writing – review & editing. NB: Investigation, Writing – review & editing. SE: Conceptualization, Writing – review & editing. KA: Investigation, Writing – review & editing. NG: Investigation, Writing – review & editing. JA: Investigation, Writing – review & editing. IP: Conceptualization, Data curation, Formal Analysis, Investigation, Writing – review & editing. TF: Conceptualization, Data curation, Formal analysis, Funding acquisition, Investigation, Methodology, Project administration, Supervision, Writing – original draft, Writing – review & editing.
