# Supplementary material for: Genotoxin-producing Salmonella enterica induces tissue-specific types of DNA damage and DNA damage response outcomes
Source: Front Immunol. 2024 Jan 11;14:1270449. doi: 10.3389/fimmu.2023.1270449 (PMC10808668; doi:10.3389/fimmu.2023.1270449)
Supplement: Supplementary file 1 [file DataSheet_1.pdf]

Supplementary Figure 1. Infection with genotoxigenic *Salmonella* induces a different host immune response in the liver and colon

Tissue sections were incubated with positive control probes to assess the technique's specificity and the tissue samples' mRNA quality. **A.** The positive probe mix consists of *Polr2a* (low expression; green), *PPIB* (medium expression; red), and *UBC* (medium/high expression; yellow) mRNA. The probe targeting the *DapB* mRNA from the *B. subtilis* was used as negative control. The panel shows representative micrographs of colon (top) and liver (bottom). **B.** Control staining to evaluate the quality of RNA upon implementation of the combined immunofluorescence-RNAscope™ protocol. Antibody anti-γH2AX coupled with the corresponding Alexa 568 secondary antibody (yellow) was combined with the positive RNAscope™ probe mix to assess low mRNA expression levels (green) or the control probe. The representative micrograph shows staining from the colon a of mouse infected with the MC1 TT strain. Inset: higher magnification of the region marked with a dotted square. Arrowheads indicate γH2AX positive cells.

Figure S1

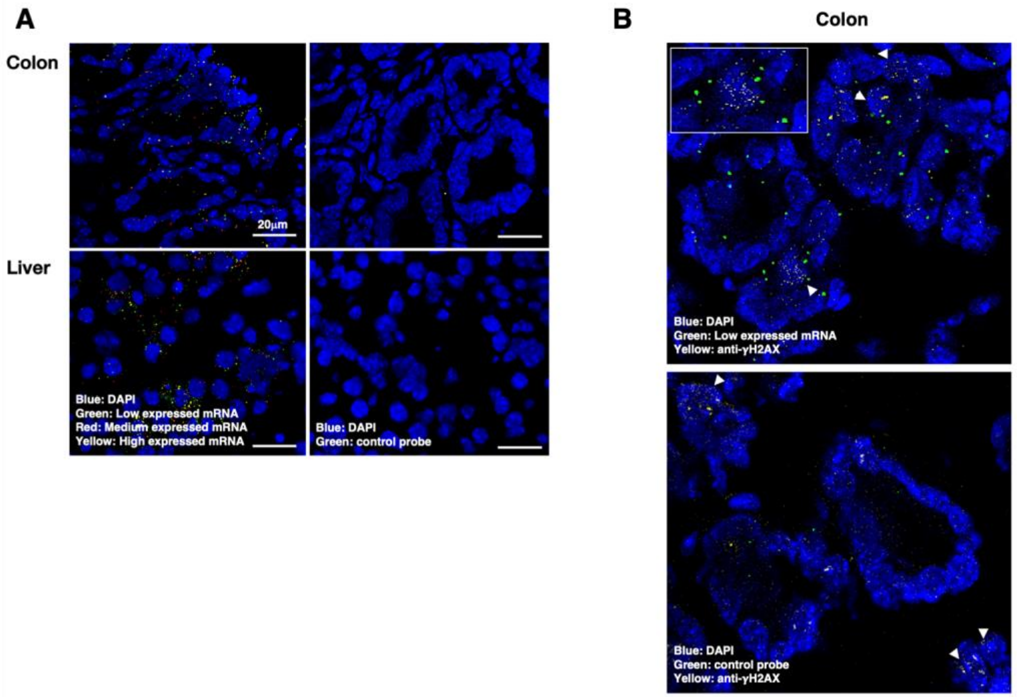

Supplementary Figure 2. Detection of cytokines, inflammasome sensors, and the flagellin subunit of *Salmonella enterica*

Mice were mock-treated with PBS (Uninf) or infected with *Salmonella* MC1 Δ*cdtB* (Δ*cdtB)* or MC1 TT (TT) for 10 days. Expression levels of the pro-inflammatory cytokine *Ifng* and the anti-inflammatory cytokine *Il10* in liver (**A**), *Ifnb1* (**B**) and *Salmonella enterica* flagellin gene *fljB* (**C**) in liver and colon were assessed by RNAscope[TM]. The data are presented as percentage positive cells in all the micrographs acquired for each mouse. A different color shade identifies individual mice within a group. * p-value ≤ 0.05; ** p-value ≤ 0.01; *** p-value ≤ 0.001; **** p-value ≤0.0001.

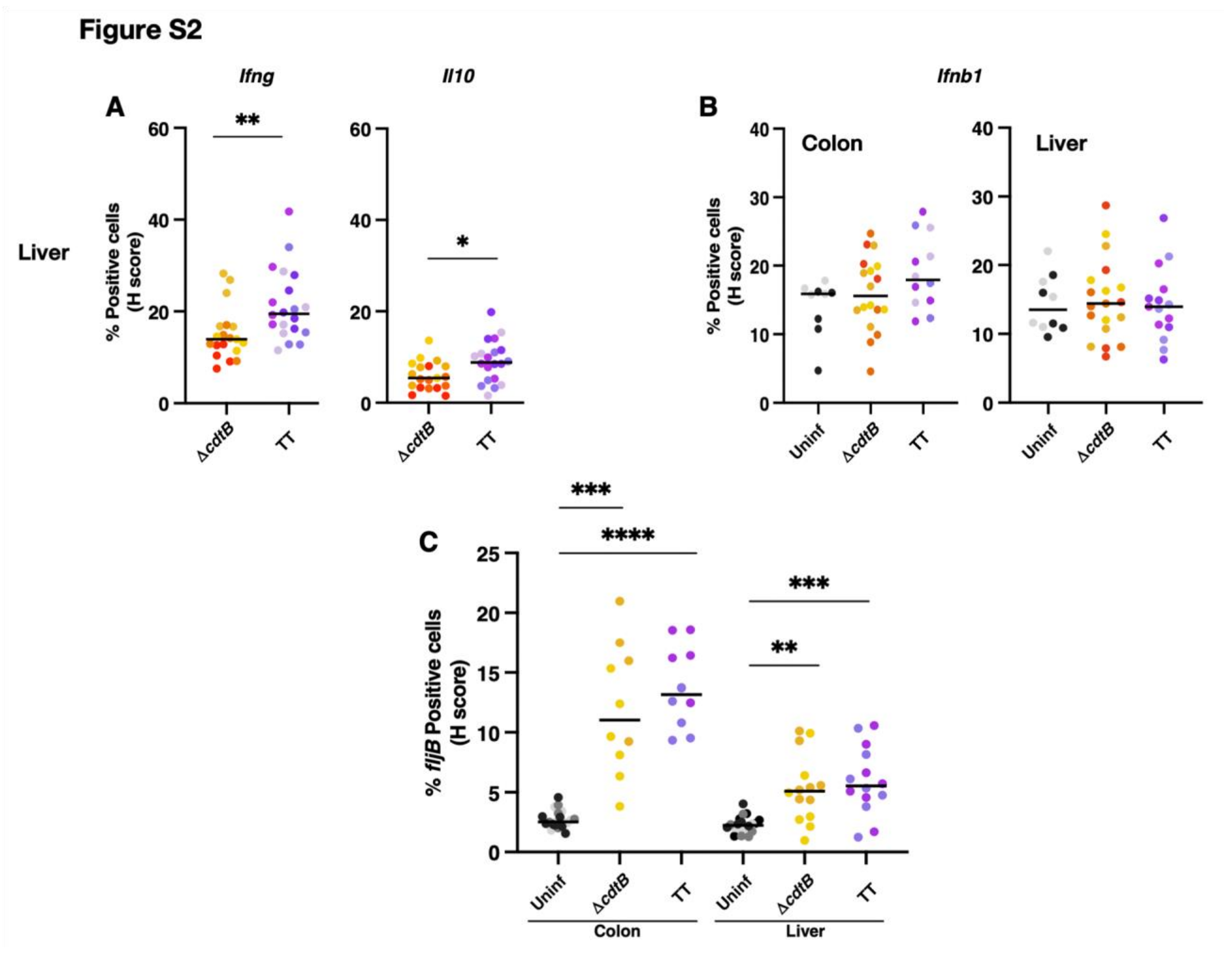

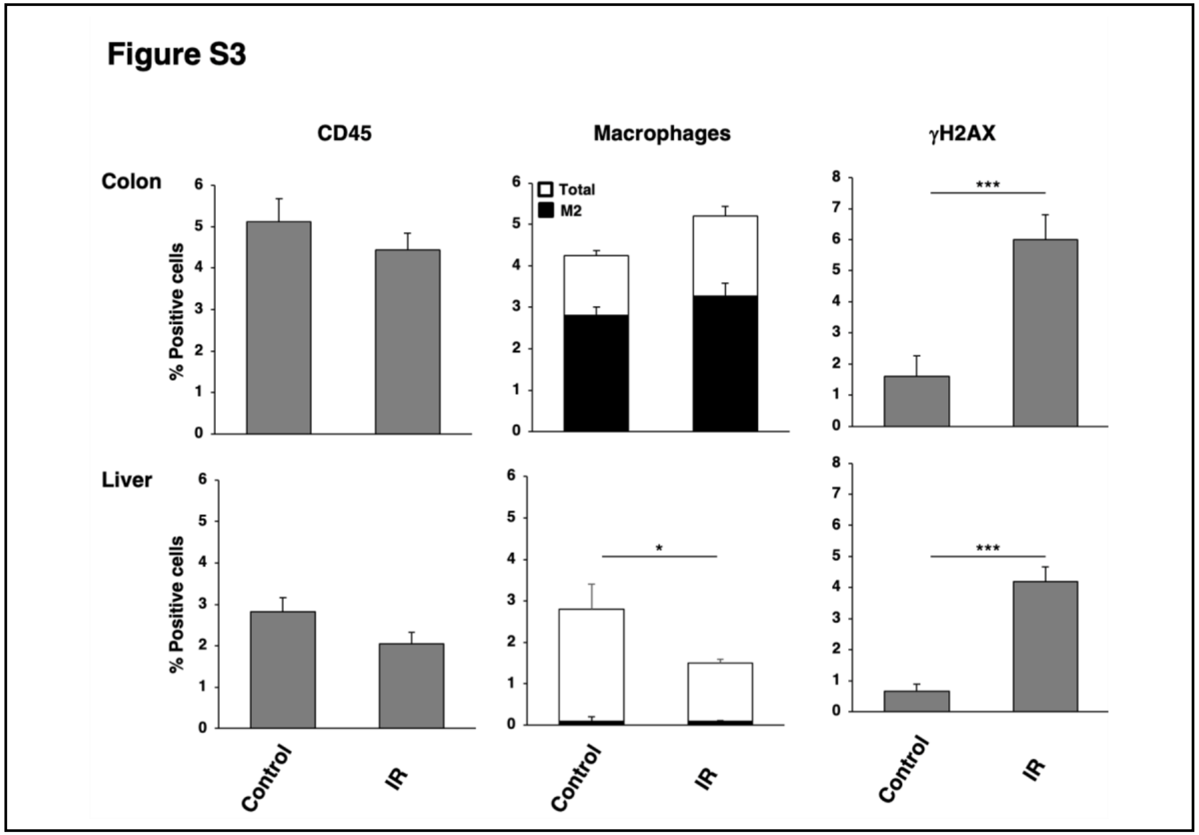

Supplementary Figure 3. Immune cell infiltration and DDR in irradiated mice

Mice were left untreated or exposed to a single dose of sublethal ionizing radiation (9Gy). The presence of immune cell types and activation of the DDR were assessed 12 hours post-treatment by immunofluorescence analysis in the colon (upper panel) and liver (lower panel).

Detection of leukocytes was performed using a rabbit anti-CD45 specific antibody. Macrophages were detected using a rat anti-F4/80 antibody for total macrophages and a goat anti-CD206 antibody for anti-inflammatory M2-like macrophages. Activation of the DDR was assessed using a rabbit anti- γH2AX specific antibody.

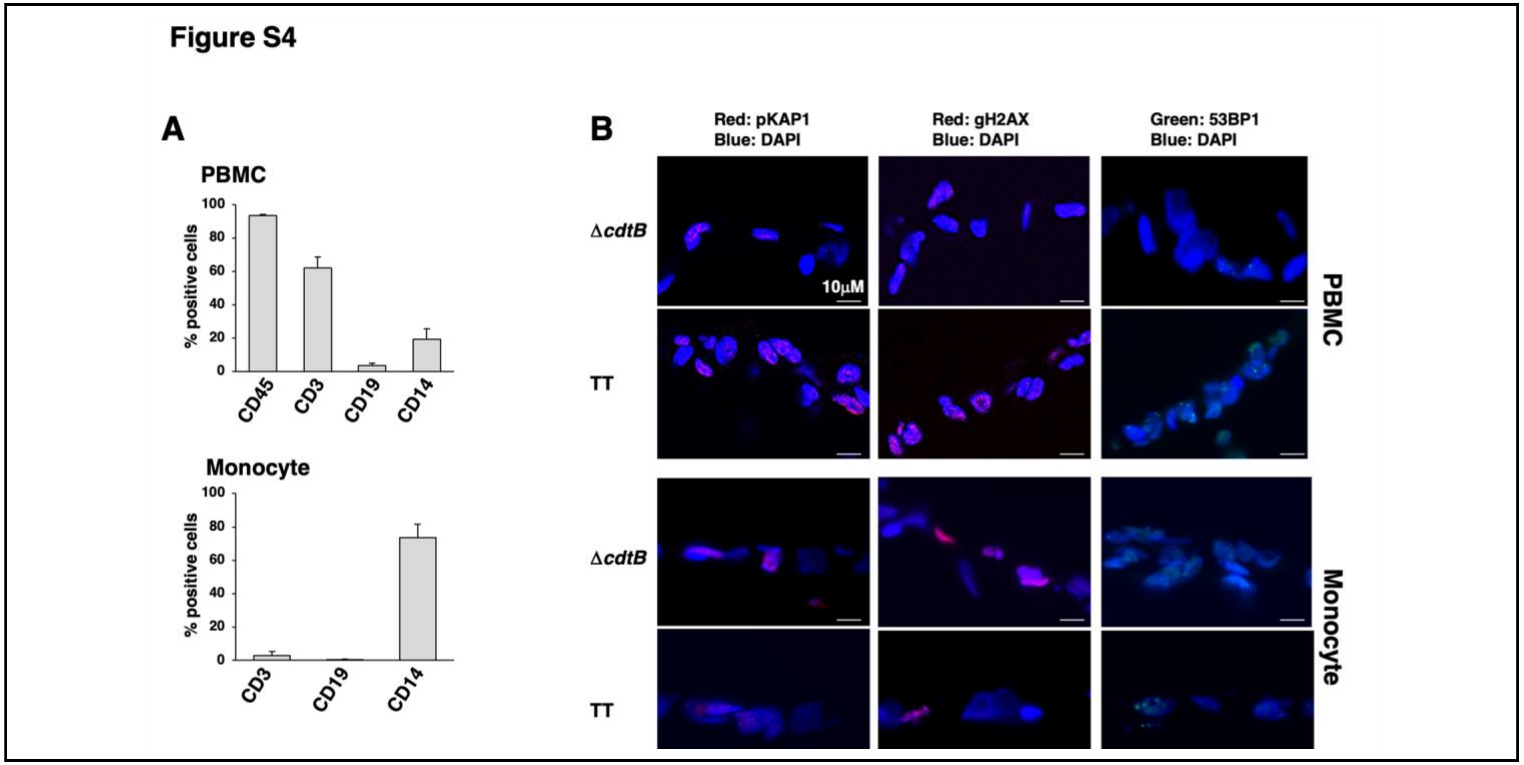

Supplementary Figure 4. Phenotypic analysis of human peripheral blood mononuclear cells

A. Upper panel: phenotypic analysis of the isolated PBMCs, indicating the presence of leukocytes (CD45 positive), T lymphocytes (CD3 positive), B lymphocytes (CD19 positive), and monocytes (CD14 positive). Lower panel: phenotypic analysis of the CD14 purified population, showing the efficiency of the purification. Mean ± SEM of 3 independent experiments. **B.** Representative fluorescence images for the activation of the DDR response in the 1CT 3D culture models established in the presence of PBMCs or purified monocytes and infected, as described in Figure 7.
